# Erratum for Bende et al., “The *Neosartorya (Aspergillus) fischeri* antifungal protein NFAP2 has low potential to trigger resistance development in *Candida albicans in vitro*”

**DOI:** 10.1128/spectrum.00611-26

**Published:** 2026-07-10

**Authors:** Gábor Bende, Nóra Zsindely, Krisztián Laczi, Zsolt Kristóffy, Csaba Papp, Attila Farkas, Liliána Tóth, Szabolcs Sáringer, László Bodai, Gábor Rákhely, Florentine Marx, László Galgóczy

## ERRATUM

Volume 13, no. 1, e01273-24, 2025, https://doi.org/10.1128/spectrum.01273-24. Due to a processing error, Fig. S2, S3, and S4 did not render properly. Figures S2, S3, and S4 are provided in this erratum.

## SUPPLEMENTAL MATERIAL

Supplemental figures spectrum.00611-26-s0001.docx Figures S2, S3, and S4.

ASM does not own the copyrights to Supplemental Material that may be linked to, or accessed through, an article. The authors have granted ASM a non-exclusive, world-wide license to publish the Supplemental Material files. Please contact the corresponding author directly for reuse.

