# Supplementary material for: Erratum for Bende et al., “The Neosartorya (Aspergillus) fischeri antifungal protein NFAP2 has low potential to trigger resistance development in Candida albicans in vitro”
Source: Microbiol Spectr. 2026 Jul 10;14(8):e00611-26. doi: 10.1128/spectrum.00611-26 (PMC13436389; doi:10.1128/spectrum.00611-26)
Supplement: Supplementary file 1 [file spectrum.00611-26-s0001.docx]

**FIG S2** Locations of detected *Candida albicans* CBS 5982 gene mutations at amino acid level as consequences of *Neosartorya* (*Aspergillus*) *fischeri* NRRL 181 antifungal protein 2 (NFAP2) or fluconazole (FLC) pressure. Conserved domains are indicated below query seq. The specific positions (amino acid number) of respective mutations in the sequence are indicated with red arrows. The amino acid alterations are indicated with colored letters. Amino acids are categorized by color based on their distinctive properties: Orange small nonpolar, green: hydrophobic, magenta: polar, red: positively charged, blue: negatively charged. CHC1: clathrin heavy chain 1, INP51: phosphoinositide 5-phosphatase, PGA58: putative glycosylphosphatidylinositol-anchored protein, PTC2: serine/threonine-specific protein phosphatase, RIM101: alkaline-responsive transcriptional regulator, UBIP: Uncharacterized biofilm-induced protein, *: termination codon, Ø: deletion. Graphical summaries were generated by Conserved Domain Search (https://www.ncbi.nlm.nih.gov/Structure/cdd/wrpsb.cgi; default options with “Standard display” result mode) using Conserved Domain Database (https://www.ncbi.nlm.nih.gov/cdd/).

**FIG S3** Scanning electron microscopy of the parental wild-type *Candida albicans* CBS 5982 (WT), *Neosartorya* (*Aspergillus*) *fischeri* antifungal protein 2-tolerant (NFAP2/4, 5, 6), and fluconazole-resistant (FLC/3, 5, 6) strains (A), and the impact of NFAP2-treatment (1 × minimum inhibitory concentration, 30 min, 30°C, 160 rpm, low-cation medium) on cell morphology (B). 2,000× magnification. Scale bars represent 10 µm.

**FIG S4** Confocal laser scanning microscopy of the parental wild-type CBS 5982 (WT), NFAP2-tolerant (NFAP2/4, 5, 6), and fluconazole-resistant (FLC/3, 5, 6) *Candida albicans* CBS 5982 strains (4 × 10^6^ cells) after treatment with the green fluorophore BODIPY-labelled NFAP2 (Bd-NFAP2, 3.125 µg ml^−1^) for 30, 60, 120, and 240 minutes (30°C, 160 rpm, low cationic medium), and co-stained with propidium-iodide (PI) for 10 minutes. Sequential scanning was done for Bd-NFAP2 (BD, green) and PI (PI, red) at 488 and 543 nm, respectively. M: merged BP, PI, and bright field pictures. Scale bars represent 10 µm. The gamma settings of the merged images have been adjusted to uniformize the tones of the backgrounds for illustrative purposes.
